# Synthesis, Complexation Properties, and Evaluation of New Aminodiphosphonic Acids as Vector Molecules for ^68^Ga Radiopharmaceuticals

**DOI:** 10.3390/molecules26082357

**Published:** 2021-04-18

**Authors:** Alesya Ya. Maruk, Valery V. Ragulin, Iurii A. Mitrofanov, Galina S. Tsebrikova, Vitaly P. Solov’ev, Alexandr S. Lunev, Kristina A. Lunyova, Olga E. Klementyeva, Vladimir E. Baulin, Galina E. Kodina, Aslan Yu. Tsivadse

**Affiliations:** 1 Department of Radiation Medical Technologies, State Research Center—Burnasyan Federal Medical Biophysical Center of Federal Medical Biological Agency, Zhivopisnaya str. 46, 123182 Moscow, Russia; amaruk@list.ru (A.Y.M.); mitrofanoff.yura@yandex.ru (I.A.M.); mr.alekslunev@gmail.com (A.S.L.); christfmbc@gmail.com (K.A.L.); klementyeva.olga@gmail.com (O.E.K.); gkodina@yandex.ru (G.E.K.); 2 Laboratory of Organophosphorus Сompounds, Institute of Physiologically Active Compounds, Russian Academy of Sciences, Severnyi proezd 1, 142432 Chernogolovka, Russia; rvalery@dio.ru (V.V.R.); mager1988@gmail.com (V.E.B.); 3 Laborotary of Novel Physicochemical Problems, Frumkin Institute of Physical Chemistry and Electrochemistry, Russian Academy of Sciences, Leninskii pr. 31/4, 119071 Moscow, Russia; solovev-vp@mail.ru (V.P.S.); tsiv@phyche.ac.ru (A.Y.T.)

**Keywords:** ^68^Ga, diphosphonate, aminodiphosphonic acid, stability constant, radiopharmaceutical, bone-seeking, inflammation, ^68^Ga-citrate

## Abstract

Two new aminodiphosphonic acids derived from salicylic acid and its phosphonic analogue were prepared through a simple and efficient synthesis. 2-[(2-Amino-2,2-diphosphono)ethyloxy]-benzoic acid **8** and 2-[(2-amino-2,2-diphosphono)ethyloxy]-5-ethyl-phenylphosphonic acid **9** were evaluated for their applicability as ^68^Ga binding bone-seeking agents. Protonation constants of **8** and **9** and stability constants of the Ga^3+^ complexes with **8** and **9** in water were determined. The stability constant of Ga^3+^ complex with fully phosphorylated acid **9** (log*K*_GaL_ = 31.92 ± 0.32) significantly exceeds stability constant of Ga^3+^ complex with **8** (log*K*_GaL_ = 26.63 ± 0.24). Ligands **8** and **9** are as effective for Ga^3+^ cation binding as ethylenediamine-*N*,*N’*-diacetic-*N*,*N’*-bis(methy1enephosphonic) acid and ethylenediamine-*N*,*N*,*N’*,*N’*-tetrakis(methylenephosphonic) acid, respectively. The labelling process and stability of [^68^Ga]Ga-**8** and [^68^Ga]Ga-**9** were studied. Both **8** and **9** readily form ^68^Ga-complexes stable to ten-fold dilution with saline. However, in fetal bovine serum, only [^68^Ga]Ga-**9** was stable enough to be subject to biological evaluation. It was injected into rats with bone pathology and aseptic inflammation of soft tissues. For [^68^Ga]Ga-**9** in animals with a bone pathology model in 60 and 120 min after injection, a slight accumulation in the pathology site, stable blood percentage level, and moderate accumulation in the liver were observed. For animals with an aseptic inflammation, the accumulation of [^68^Ga]Ga-**9** in the pathology site was higher than that in animals with bone pathology. Moreover, the accumulation of [^68^Ga]Ga-**9** in inflammation sites was more stable than that for [^68^Ga]Ga-citrate. The percentage of [^68^Ga]Ga-**9** in the blood decreased from 3.1% ID/g (60 min) to 1.5% ID/g (120 min). Accumulation in the liver was comparable to that obtained for [^68^Ga]Ga-citrate.

## 1. Introduction

Generator-produced positron-emitting ^68^Ga (T_1/2_ = 67.71 min) is one of the most promising radionuclides [1,2]. ^68^Ga radiopharmaceuticals are used for diagnostics of neuroendocrine tumors and prostate cancer, or for visualization of infection and inflammation [3,4,5]. Many studies are devoted to the search of osteotropic ^68^Ga radiopharmaceuticals for imaging bone metastases during the early stages [6,7,8,9]. Phosphonate ligands are commonly used for this purpose as they work with other radionuclides (^99m^Tc, ^111^In, ^153^Sm, ^188^Re) [10,11]. 1-Amino-1,1-diphosphonic acids having fine complexation properties are of interest as ligands for radiopharmaceuticals. These compounds are structural diphosphonic analogues of physiologically important aminocarboxylic acids and have low toxicity [12,13]. Besides, they can be easily prepared by the addition of two phosphorous acid molecules to a nitrile group.

**Figure 1:**
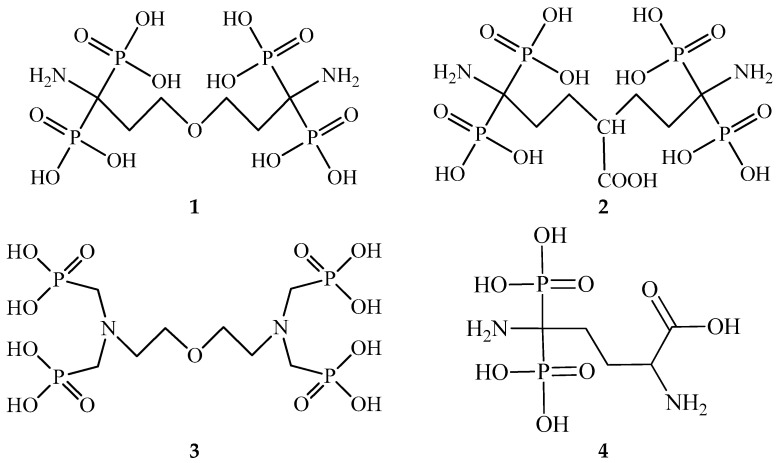
Structures of phosphonic acids mentioned in this work.

Recently, we studied two ^68^Ga complexes with organic ligands containing aminodiphosphonic groups (1,7-diamino-4-oxaheptane-1,1,7,7-tetraphosphonic (**1,** Figure 1) and 1,7-diamino-4-hydroxycarbonylheptane-1,1,7,7-tetraphosphonic acids (**2, **Figure 1)) as potential bone-seeking agents [14,15]. Compounds **1** and **2** are closely related to the oxa-bys-ethylenenitrile tetra(methylene phosphonic acid) (**3,** Figure 1), which is a component of the widely used in Russia [^153^Sm]samarium oxabifore therapeutic radiopharmaceutical [16]. The osteotropic properties of the previously obtained ^188^Re-complex with 2,5-diamino-5,5-diphosphonovaleric acid (**4,** Figure 1) showed that the combination of aminodiphosphonic and α-amino acid moieties in one molecule is a new way to develop radiopharmaceuticals [17]. In this regard, it is of interest to search for new ligands for radiopharmaceuticals by combining aminodiphosphonic fragment and molecules with known complexation and medicinal properties. Salicylic acid may be a promising object for further functionalization [18]. Derivatives of (2-hydroxyphenyl)phosphonic acid are poorly studied analogues of salicylic acid in which the carboxyl group is replaced by a phosphonic moiety. This can significantly affect both the complexation and biological properties. It was found that (2-hydroxy-5-ethylphenyl)phosphonic acid is of interest as a potential non-steroidal anti-inflammatory drug [19].

**Figure 2:**
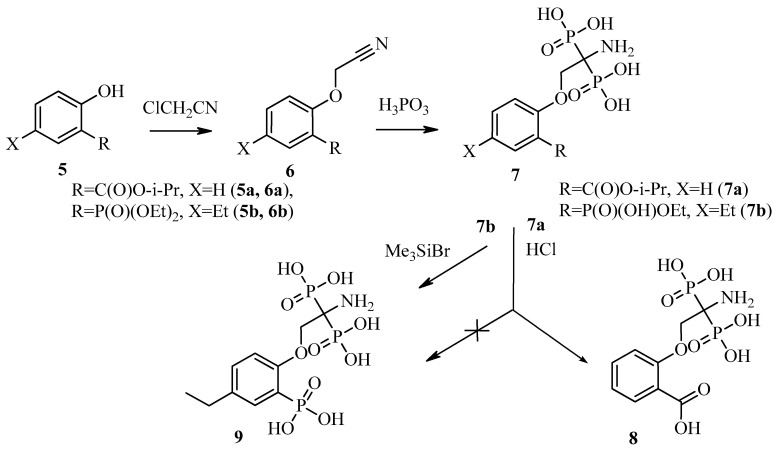
General procedure for the synthesis of 2-[(2-amino-2,2-diphosphono)ethyloxy]-benzoic acid (**8**) and 2-[(2-amino-2,2-diphosphono)ethyloxy]-5-ethyl-phenylphosphonic acid (**9**).

In this work, two new ligands were synthesized and evaluated for their applicability as ^68^Ga binding bone-seeking agents: 2-[(2-amino-2,2-diphosphono)ethyloxy]-benzoic acid (**8**) and 2-[(2-amino-2,2-diphosphono)ethyloxy]-5-ethyl-phenylphosphonic acid (**9**) (Figure 2). Protonation constants of **8** and **9** and stability constants of the Ga^3+^ complexes with **8** and **9** in water were determined. New compounds were labelled with ^68^Ga. Particular aspects of the labelling process and stability of [^68^Ga]Ga-**8** and [^68^Ga]Ga-**9** were studied. The biodistribution of [^68^Ga]Ga-**9** was studied in animals with bone pathology and aseptic inflammation of soft tissues.

## 2. Results and Discussion

### 2.1. Synthesis

Two new ligands known as 2-[(2-amino-2,2-diphosphono)ethyloxy]-benzoic acid (**8**) and 2-[(2-amino-2,2-diphosphono)ethyloxy]-5-ethyl-phenylphosphonic acid (**9**) were prepared using a simple and efficient synthesis.

Salicylic acid and its phosphonic analogue were functionalized by alkylation of phenolic oxygen using monochloroacetonitrile. The obtained nitrile group was easily converted to the aminodiphosphonic group by the addition of two phosphorous acid molecules, resulting in acids **8** and **9** (Figure 2). Previously, we used this reaction of phosphorous acid addition to a nitrile group in order to obtain a number of aminodiphosphonic acids, promising as physiologically active compounds [17,20]. Molecules **8** and **9** contain a fragment of salicylic acid **5a** or its phosphonic analogue **5b** and a structural diphosphonic isostere of serine, linked by an ether bond.

The starting compounds for the synthesis of new aminodiphosphonic acids **8** and **9** were isopropyl salicylate **5a** (R = COO-iPr, X = H) and diethyl (2-hydroxy-5-ethylphenyl)phosphonate **5b** (R = P(O)(OEt)_2_, X = Et). The alkylation of phenols **5a** and **5b** with monochloroacetonitrile gave nitriles **6a** and **6b**, respectively (Figure 2). The following addition of two molecules of phosphorous acid in dioxane in the presence of phosphorus tribromide led to aminodiphosphonic acids **7a** and **7b** (as monoethyl ester). The desired water-soluble form of **8** was obtained by hydrolysis of isopropyl ester **7a** with 6N hydrochloric acid. An attempt to obtain phosphonic analogue **9** in a similar way led to the break of the phosphorus-carbon bond and the loss of the ortho-phosphonic fragment. Acid **9** was successfully obtained after treatment of ethylphosphonate **7b** with trimethylsilyl bromide in an acetonitrile solution (Figure 2).

According to ^13^C and ^31^P NMR, molecule **7a** is likely a mixture of two conformers having a different orientation (syn:anti~1:4) of the aminodiphosphonic fragment and the isopropyl radical relative to the aromatic ring. The spectral data demonstrate the magnetic equivalence of phosphorus atoms of the aminodiphosphonic fragment. According to ^31^P NMR, molecule **7b** is a mixture of two conformers with a ratio of ~3:1 (see Materials and Methods: signals of a minor conformer are marked by an asterisk (*)). The ^31^P NMR spectrum of each conformer includes two signals with a ratio of 1:2 from arylphosphonic and aminodiphosphonic fragments. Thus, phosphonate **7b** exists in the form of a mixture of two conformers with the syn-orientation and anti-orientation of the aminodiphosphonic fragment and the ester fragments of the phosphonic function relative to the aromatic ring, similarly to aminodiphosphonic acid **7a**. The presence of the second form in the case of acid **9** is likely determined by the syn-orientation or anti-orientation of the methyl group in the ethyl radical and the aminodiphosphonic fragment. Perhaps, this explains the fact that the ratio of conformers, in this case, is ~1:10.

### 2.2. Stability Constants of Gallium(III) Complexes with 8 and 9

One of the most important criteria for the suitability of ligands as components of radiopharmaceuticals is the stability of their complexes with radionuclides [3,21,22]. For this, the protonation constants of the ligands and the stability constants of their complexes with Ga^3+^ were determined.

The protonation constants of **8** (H_5_L) and **9** (H_6_L) were determined at 298 К and ionic strength of *I* = 0.1 M KCl. Stepwise equilibrium constants of acids are given in Table 1. Full constants are given in Table S1 (see Supplementary Materials). The values of the stepwise constants of **8** and **9** are in good agreement with data for other aminodiphosphonic acids [23]. The first three protonation constants (no. 1–3, Table 1) of **8** are similar to the corresponding constants of (aminoethylene)diphosphonic acid (AEDP), which is essentially a fragment of **8**.

The complexation reactions of **8** and **9** with Ga^3+^ in water at 298 К and ionic strength of *I* = 0.1 M KCl is well described by the model, which includes the complexation of Ga^3+^ with a deprotonated ligand and addition to Ga^3+^ of one to three (**8**) or one to four (**9**) protons besides the ligand (Table 2; full constants are given in Table S2, see Supplementary Materials). Stability constant of the Ga^3+^ complex with deprotonated ligand **9** (log*K*_GaL_ = 31.92 ± 0.32) significantly exceeds the corresponding stability constant of the Ga^3+^ complex with **8** (log*K*_GaL_ = 26.63 ± 0.24) (Table 2). This is very consistent with the fact that acid **9** has the more active P(O)(OH)_2_ group for the complexation instead of the COOH group of **8**. This result suggests that ligand **9** is preferable over ligand **8** as a radiopharmaceutical component. The stability constants of the gallium(III)-deprotonated ligand complexes for **8** and **9** are similar to the stability of the complexes for ethylenediamine-*N*,*N’*-diacetic-*N*,*N’*-bis(methylenephosphonic) acid (EDDADPO) (log*K*_GaL_ = 26.82) and for the ethylenediamine-*N*,*N*,*N’*,*N’*-tetrakis(methylenephosphonic) acid (EDTPO) (log*K*_GaL_ = 31.83) [25] (Table 2 and Table S2 in Supplementary Materials). Ligands **8** and **9** are effective ligands for gallium(III) as EDDADPO and EDTPO, respectively. Since [^68^Ga]Ga-EDTPO showed promising results in μ-PET studies, we supposed that a study of osteotropic properties of **8** and **9** is of interest.

### 2.3. Radiolabelling and Stability

Both **8** and **9** ligands readily form complexes with ^68^Ga at room temperature when using ligand concentrations >5 mM and pH 5 ± 2 as confirmed by radio-TLC. Both [^68^Ga]Ga-**8** and [^68^Ga]Ga-**9** obtained in this way were stable for ten-fold dilution with saline. However, when ten-fold diluted with fetal bovine serum, up to 90% and 50% of [^68^Ga]Ga-**8** and [^68^Ga]Ga-**9** correspondingly undergo decomposition. Elevation of **9** concentration to 20 mM and raising reaction temperature to 95 °C (30-min reaction time) allowed us to achieve 60–90% stability in a 2 hr interval. This approach did not work out for [^68^Ga]Ga-**8,** and regardless of the concentration of **8** in the mixture, temperature, and reaction time, the stability of [^68^Ga]Ga-**8** was very low. These results are in good agreement with the stability constants of gallium(III) complexes with **8** and **9** in water. Thus, it is once more affirmed that phosphonic groups are significantly more favorable for M^3+^ radiometals binding over carboxylic groups [26]. In the frame of this study, we decided to carry out further detailed studies using [^68^Ga]Ga-**9**.

**Figure 3:**
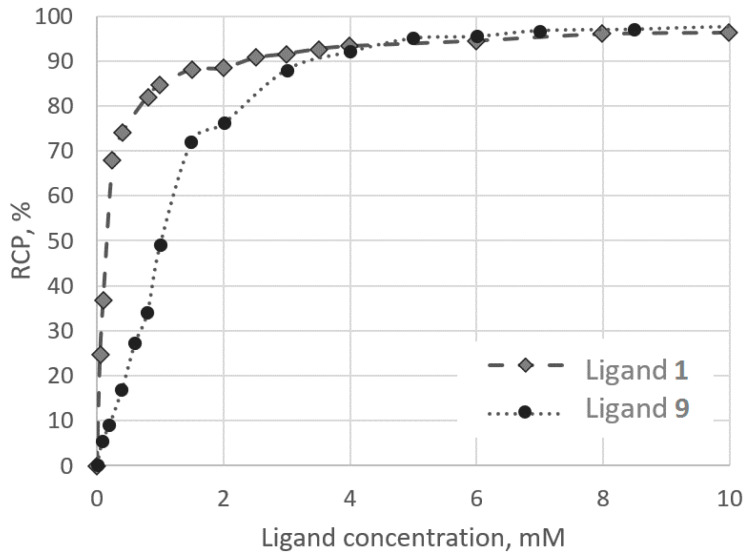
Ligand concentration—labelling reaction yield dependencies for [^68^Ga]Ga-**1** and [^68^Ga]Ga-**9**.

Figure 3 shows the ligand concentration—labelling reaction yield dependence for the ligand **9** and previously studied 1,7-diamino-4-oxaheptane-1,1,7,7-tetraphosphonic acid (**1**) [10]. The conditions of ^68^Ga-labelling for both compounds were the same: рН 6, acetate concentration of 0.2 M, 25 °C, and 15 min reaction time. Ligand **1** contains two amidiphosphonic groups separated by a five-atoms ether chain with a weak donor oxygen atom. Ligand **9** contains one amidiphosphonic group and one phosphonic group in orto-position to it. According to potentiometric studies, ligand **1** forms one more protonated complex than ligand **9**. The labelling reaction yield at low ligand concentration for [^68^Ga]Ga-**1** is higher than that for [^68^Ga]Ga-**9**. Clearly, the structures of the Ga^3+^ complexes of these ligands differ significantly. This can be attributed to the fact that amidiphosphonic groups may be more effective for ^68^Ga binding than phosphonic groups. Another possible explanation may be the orto-position of the phosphonic group in **9** preventing the amidiphosphonic group from realizing its full binding potential toward ^68^Ga.

**Figure 4:**
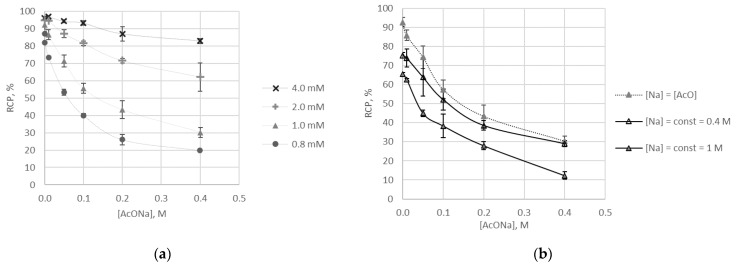
The effect of buffer component concentrations on the labelling reaction yield for [^68^Ga]Ga-**9**: (**a**) at Na^+^ concentration being equal to that of acetate ion and at various concentrations of ligand **9**, (**b**) comparison of the effects of dynamic and constant Na^+^ concentration at the same 1 mM concentration of **9**.

The influence of the buffering agent type on the labelling reaction yield was analysed before for [^68^Ga]Ga-**1** [14]. The influence of acetate concentration on the labelling reaction yield was demonstrated for DOTA-conjugated molecules [27]. Here, the effect of acetate concentration was studied using four ligand **9** concentrations at a constant pH 5.9 ± 0.4. To observe the effect of ligand **9**, the following concentrations were chosen: 0.8, 1.0, 2.0, and 4.0 mM. Results are presented in Figure 4a. In the case of [^68^Ga]Ga-**9**, there is a distinct correlation: the lower the acetate concentration is, the higher is the labelling reaction yield with maximal yield achieved in the absence of acetate. This correlation becomes less significant with increasing ligand concentration and becomes statistically insignificant (*p* > 0.05) at 5 mM of **9**. In addition to the results [14], we carried out similar experiments with [^68^Ga]Ga-**1** and found no statistically significant correlation even at a concentration of **1** in the reaction mixture being as low as 0.2 mM. Thus, there are three patterns of acetate concentration influencing the labelling reaction yield.

- no correlation for [^68^Ga]Ga-**1**;
- correlation with an extremum point for [^68^Ga]Ga-DOTA-conjugates (0.3 M of acetate corresponds to maximum labelling reaction yield) [27];
- continuous dependence for [^68^Ga]Ga-**9** (with maximum labelling reaction yield corresponding to the minimal concentration of acetate).

Gallium is known to form weak acetate complexes [28,29]. With log*K* = 3.7 [30], acetate is not able to compete with **9** for gallium binding. There is a possibility of a ternary Ga-**9**-OAc complex (or complexes) formation similar to that described for copper [18]. This possibility should be a subject of a separate study. In this study, acetate ion was added to reaction mixtures exclusively in the form of sodium acetate. The additional concentration of sodium in the reaction mixtures due to using NaOH for pH adjusting was ≤ 0.003 M. This suggests that sodium concentration in studied samples is virtually equal to that of acetate. To evaluate the influence of Na^+^ on the labelling process, additional experiments with constant Na^+^ concentrations were carried out. For this purpose, calculated amounts of NaCl were added to reaction mixtures at pH 5.7 ± 0.7 and 1 mM of **9**. The comparison of the effects of dynamic and constant sodium concentration on the labelling reaction yield is presented in Figure 4b. At constant Na^+^ concentration of 0.4 M, obtained RCP (radiochemical purity) values were consistently lower than those obtained at dynamic Na^+^ concentration. However, the differences were mostly statistically insignificant (*p* > 0.05). Still, it is reasonable to assume that the acetate ion is the component responsible for the changes demonstrated in Figure 4a. The changes of water structure induced by an Na^+^ presence were observed in 1 M sodium chloride and sodium acetate solutions [31]. The examination of this sodium concentration in our experiments resulted in much lower RCP values as compared with corresponding samples with dynamic Na^+^ concentration (*p* < 0.05, Figure 4b). Thus, it is clear that Na^+^ itself has an impact on the Ga-**9** complex formation process.

**Figure 5:**
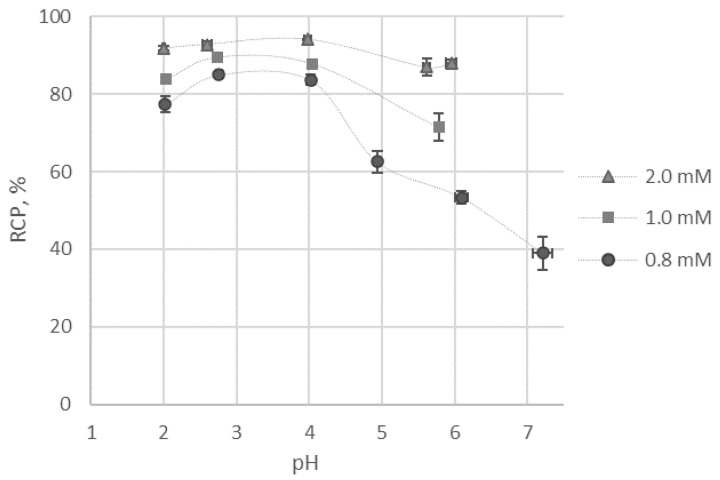
The effect of pH on the labelling reaction yield for [^68^Ga]Ga-**9**.

Finally, taking into account previous results, the effect of pH on the labelling reaction yield was studied using reaction mixtures with 50 mM of acetate and 0.8, 1.0, and 2.0 mM of ligand **9**. A maximal reaction yield was observed for the samples at pH 3–4 (Figure 5), which is consistent with the data observed for ^68^Ga [27,32]. According to the calculations based on the obtained stability constants, the protonated complex GaH_4_L^+^ of ligand **9** dominates in this pH range.

### 2.4. Biodistribution of [^68^Ga]Ga-9

In Table 3, the biodistribution data of [^68^Ga]Ga-9 and [^68^Ga]Ga-acetate in animals with fractures are presented. Non-target biodistribution pathways and bone pathology uptake for [^68^Ga]Ga-9 are comparable to those of [^68^Ga]Ga-1 and [^68^Ga]Ga-2 [14]. The pathology site/intact bone ratio for [^68^Ga]Ga-9 is inferior to that of [^68^Ga]Ga-oxa-bis-ethylenenitrile tetra(methylene phosphonic acid) (3) [33] and even to that of [^68^Ga]Ga-acetate studied in this experiment (Table 3). Moreover, [^68^Ga]Ga-3 uptake in blood, liver, intestine, and kidneys is lower than that of [^68^Ga]Ga-9. Likely, it depends on the different stability of ^68^Ga-labelled complexes in vivo and requires additional research.

Since fracture healing may be accompanied by an inflammatory process, animals with an aseptic inflammation model were also studied. In Table 4, the data on the biodistribution of [^68^Ga]Ga-9 in animals with aseptic inflammation are presented along with data for [^68^Ga]Ga-citrate, which is known to have an inflammation imaging potential [34]. During the comparison of biodistribution dynamics, it was found that [^68^Ga]Ga-9 can be a potential agent for aseptic inflammation imaging more promising than [^68^Ga]Ga-citrate. Activity in blood 120 min after injection in comparison to a 60-min time point decreases three and two times for [^68^Ga]Ga-9 and [^68^Ga]Ga-citrate, respectively. [^68^Ga]Ga-9 pathology site/muscular tissue ratio is almost constant during the time of observation. It will allow imaging pathology foci 1 h after i.v. injection (for [^68^Ga]Ga-citrate—2 h).

## 3. Materials and Methods

### 3.1. Synthesis

The progress of the reactions was monitored by ^31^P NMR spectroscopy. All chemicals and solvents were purchased from Acros Organics (Acrus, Moscow, Russia) and Alfa Aesar (Reakor, Moscow, Russia). The ^1^H, ^31^P, and ^13^C NMR spectra were recorded on a Bruker DPX-200 spectrometer (Billerica, MA, USA) at 200.13, 81.0, and 50.04 MHz, respectively. Chemical shifts *δ* are given in ppm and coupling constants *J* are given in Hz. Melting points are determined on a Boetius PHMK-05 device or in the block in an open glass capillary. Chromatographic analysis was carried out for some of the compounds on LC/MSD Agilent 1100 mass spectrometer (Santa Clara, CA, USA) with DAD, ELSD, and single quadrupole mass-selective detector with ionization by electrospray. For some of the acids, elemental analysis was also performed.

The starting isopropyl salicylate **5a** (R = COO-iPr, X = H) was purchase from Aldrich. Diethyl (2-hydroxy-5-ethylphenyl)phosphonate **5b** (R = P(O)(OEt)_2_, X = Et, Figure 1) was synthesized, according to Reference [19].

**General synthesis of 6a, b**. Sodium hydride (20 mmol) (55% suspension in paraffin) was added in small portions to the mixture of phenol **5a** or **5b** (20 mmol) in 35 mL of dry dioxane at room temperature. The mixture was stirred for 0.5 h, then chloroacetonitrile (20 mmol) was added, and the formed reaction mass was boiled by stirring for 8 h. The reaction mixture evaporated in vacuo and 50 mL of water was added to the residue. The formed solution was acidified by HCl to pH~1 and the mixture was extracted with chloroform (3 × 25 mL). The organic extract was washed with water (3 × 25 mL) and evaporated in vacuo. Nitriles **6a, b** were isolated by vacuum distillation of the residue.

**Isopropyl 2-(cyanomethyloxy)-benzoate 6a.** Сolorless oily liquid. B.p. 117–119 °C (1.1 mm). Yield 71%. ^1^H NMR (200 MHz, CDCl_3_, δ, ppm): 1.38 d (6Н, CH(CH_3_)_2_, ^3^*J*_НН_ 6.1 Hz), 4.86 s (2H, OCH_2_CN), 5.25 m (1Н, CH(CH_3_)_2_), 7.15 m (1Н, arom.), 7.54 m (2Н, arom.), 7.85 m (1Н, arom.). ^13^С NMR (50.3 MHz, CDCl_3_, δ, ppm): 21.84 (CH_3_), 55.84 (CH_2_O), 68.76 (CHO), 115.06 (CN), 116.78 (C_ar._), 123.10 (C_ar._-COO), 123.69 (C_ar._), 131.92 (C_ar._), 133.44 (C_ar._), 155.99 (C_ar._-O), 164.82 (COO).

**Diethyl 2-(cyanomethyloxy)-5-ethyl-phenylphosphonate 6b.** Сolorless oily liquid. B.p. 164–166 °C (0.8 mm). Yield 55%. ^1^H NMR (200 MHz, CDCl_3_, δ, ppm): 1.19 t (3H, CH_3_, ^3^*J*_НН_ 7.7 Hz), 1.31 t (6H, 2CH_3_, ^3^*J*_НН_ 7.2 Hz), 2.61 q (2H, CH_2_, ^3^*J*_НН_ 7.7 Hz), 4.12 dq (4H, 2CH_2_O, ^3^*J*_НН_ 7.2 Hz, ^4^*J*_PН_ 2.1 Hz), 4.83 s (2H, CH_2_O), 7.00 m (1H, arom.), 7.33 m (1H, arom.), 7.67 m (1H, arom.). ^13^С NMR (50.3 MHz, CDCl_3_, δ, ppm): 15.44, 16.30 d (^3^*J*_РOCC_ 6.2 Hz), 27.86, 54.80, 62.27 d (^2^*J*_РOC_ 5.5 Hz), 114.23 (C_ar._) d (^3^*J*_РC_ 9.9 Hz), 114.95 (CN), 118.51 (C_ar._) d (^1^*J*_РC_ 187.0 Hz), 133.57 (C_ar._), 134.71 (C_ar._) d (^2^*J*_РC_ 6.9 Hz), 139.37 (C_ar._) d (^3^*J*_РC_ 13.9 Hz), 156.09 (C_ar._-O). ^31^P NMR (81.0 MHz, D_2_O, δ, ppm): 17.1.

**General synthesis of 7a, b.** Phosphorus tribromide (20 mmol) was slowly added dropwise at 5 °C to a mixture of nitrile **6a, b** (10 mmol) and dry phosphorous acid (20 mmol) in 15 mL of dioxane. Then the reaction mixture was stirred at room temperature for about 10 h, evaporated in vacuo, and 10 mL of dioxane was added to the residue. After the formation of a gelatinous residue, dioxane was decanted, the procedure was repeated, and 30 mL of acetic acid was added to the residue with cooling and stirring. The resulting mixture was stirred for about an hour and co-evaporated with 30 mL of toluene. Water (10 mL) dropwise was added to the oily residue and left overnight without stirring. The resulting white powder was washed with a small amount of water (3 × 3 mL) and then with ethanol, and aminodiphosphonic acids **7a** and **7b** was isolated with yields 63–64%.

**Isopropyl 2-[(2-amino-2,2-diphosphono)ethyloxy]-benzoate 7a.** White solid. Yield 63% M.p. 223–225 °C (with decomp.). ^1^H NMR (200 MHz, D_2_О, δ, ppm): 1.31 d (6Н, 2СН_3_, ^3^*J*_НН_ 6.1 Hz), 4.55 d (1Н, one of CH_2_O, ^3^*J*_PН_ 7.9 Hz), 4.60 d (1Н, second of CH_2_O, ^3^*J*_PН_ 8.5 Hz), 5.16 m (1H, OCH), 6.95–7.20 m (2H, arom.), 7.50–7.62 m (1H, arom.), 7.80–7.90 m (1H, arom.). ^13^С NMR (50.3 MHz, D_2_О, δ, ppm): 21.11, *23.35 (СН_3_), 57.47 t (^1^*J*_РC_ 116.6 Hz) (P_2_СN), *69.38, 69.68 (CH_2_O), 70.39 (OCH), *114.46, 114.84, *119.34, 119.68, 121.27, 121.73, *131.60, 131.85, 134.95, 157.83 (C = O), *167.64 (C = O). ^31^P NMR (81.0 MHz, D_2_O, δ, ppm): 10.4, * 14.1 (4:1). (*—hereinafter minor conformers). Found, %: C 37.42, 37.35; H 5.13, 5.33. C_12_H_19_NO_9_P_2_. Calculated, %: C 37.61, H 5.00. LCMS calcd for C_12_H_19_NO_9_P_2_: 383.2. Found 384.4 (protonated form).

**Monoethyl 2-(2-amino-2,2-diphosphono)ethyloxy-5-ethyl-phenylphosphonate 7b.** White solid. Yield 64% M.p.: 205–208 °C (with decomp.). ^1^H NMR (200 MHz, DMSO-d6 + drop TFA, δ, ppm): 1.12 t (3Н, СН_3_, ^3^*J*_НН_ 7.3 Hz), 1.28 t (3Н, СН_3_, ^3^*J*_НН_ 6.7 Hz), 2.55 q (2Н, СН_2_, ^3^*J*_НН_ 7.3 Hz), 4.08 q (2H, СН_2_О, ^3^*J*_НН_ 7.3 Hz), 4.48 m (broad) (2Н, СН_2_СР), 6.90–7.05 m (1Н, СН arom.), 7.27–7.40 m (2Н, СН arom.). ^1^H NMR (200 MHz, D_2_О, δ, ppm): 1.10 t (3Н, СН_3_, ^3^*J*_НН_ 7.3 Hz), 1.15 t (3Н, СН_3_, ^3^*J*_НН_ 7.3 Hz), 2.54 q (2Н, СН_2_, ^3^*J*_НН_ 7.3 Hz), 3.78 q, and 4.09*q (2H, СН_2_, ^3^*J*_НН_ 7.3 Hz), 4.40 d (1Н, one of СН_2_СР, ^2^*J*_РН_ 9.8 Hz), 4.46 d (1Н, the second of СН_2_СР, ^2^*J*_РН_ 9.8 Hz), 6.87–7.15 m (1Н, СН arom.), 7.20–7.55 m (2H, CH arom.). ^13^С NMR (50.3 MHz, D_2_О, δ, ppm): 15.22, 15.83 d (^3^*J*_РC_ 6.5 Hz), 27.42, 58.16 t (^1^*J*_РC_ 120.2 Hz) (P_2_СN), and *58.26 t (^1^*J*_РC_ 121.5 Hz) (P_2_СN), 61.93 d (^2^*J*_РOC_ 5.0 Hz), and *64.28 d (^2^*J*_РOC_ 5.8 Hz), 68.99 and *69.43 (OCH_2_CN), 112.32 d (^3^*J*_РC_ 8.4 Hz), and *112.82 d (^3^*J*_РC_ 8.1 Hz), 118.62, 121.86, 122.13, 127.21 d (^1^*J*_РC_ 186.3 Hz), *131.56 (^3^*J*_РC_ 6.5 Hz) and 132.20 (^3^*J*_РC_ 6.9 Hz), 132.64, 137.78 d (^3^*J*_РC_ 12.7 Hz), *156.73, and 156.90. ^31^P NMR (81.0 MHz, DMSO-d_6_, δ, ppm): (10.38 + *10.90) / (*14.83 + 16.30) = 2/1. ^31^P NMR (81.0 MHz, D_2_O, δ, ppm): (*11.65 + 13.03) / (*10.76 + 14.76) = 2/1. Found, %: P 21.73, 21.85. C_12_H_22_NO_10_P_3_. Calculated, %: P 21.45.

**2-[(2-Amino-2,2-diphosphono)ethyloxy]-benzoic acid 8.**

Solution of isopropyl ether **7a** (1.9 g, 5 mmol) in a 10 mL of 6N HCl was refluxed for 5 h. Acid **8** was isolated after evaporation of the reaction mixture and crystallization of the residue from aqueous ethanol. White solid. Yield 1.3 g (76%). M.p.: 243–244 °C (with decomposition). ^1^H NMR (200 MHz, D_2_О + NaOD, pH~10, δ, ppm): 4.27 t (2Н, СН_2_О, ^3^*J*_РН_ 12.2 Hz), 6.85–6.98 m (1Н, СН arom.), 7.08–7.20 m (1Н, СН arom.), 7.24–7.38 m (2H, 2СН arom.). ^13^С NMR (50.3 MHz, D_2_О + NaOD, pH~10, δ, ppm): 58.06 t (^1^*J*_РC_ 120.7 Hz), 72.98, 115.30, 121.50, 128.50, 129.38, 130.90, 155.87, 177.15 (C = O). ^31^P NMR (81.0 MHz, D_2_O + NaOD, δ, ppm): 9.85 (pH 5), 19.60 (pH 10). Found, %: C 31.41, 31.35, H 4.04, 3.96, P 18.23, 18.05. C_9_H_13_NO_9_P_2_. Calculated, %: C 31.69, H 3.84, P P18.16. LCMS calcd for C_9_H_13_NO_9_P_2_: 341.2. Found 342.2 (protonated form).

**2-[(2-Amino-2,2-diphosphono)ethyloxy]-5-ethyl-phenylphosphonic acid 9**. To a solution of 0.73 g (1.6 mmol) monoethyl ester of phosphonic acid **7b** in 7 mL of dry acetonitrile 0.21 mL (1.6 mmol) of trimethylsilyl bromide was added. The mixture was gradually heated with stirring and boiled for 5 h. The reaction mixture was evaporated in vacuo. The residue was twice evaporated with 5 mL of water and evaporated to dryness. The resulting solid was washed with water and then with ethanol. Acid **9** was isolated after crystallization of the residue from aqueous ethanol. White solid. Yield 68%. M.p.: 239–240 °C (with decomposition). ^1^H NMR (200 MHz, D_2_О, δ, ppm): 1.11 t (3Н, СН_3_, ^3^*J*_НН_ 7.3 Hz), 2.55 q (2Н, СН_2_, ^3^*J*_НН_ 7.3 Hz), 4.52 d (1Н, one of СН_2_О, ^3^*J*_РН_ 9.8 Hz), 4.55 d (1Н, the second of СН_2_О, ^3^*J*_РН_ 9.3 Hz), 6.90–7.05 m (1Н, СН arom.), 7.27–7.55 m (2Н, 2СН arom.). ^13^С NMR (50.3 MHz, D_2_О, δ, ppm): 15.30, 27.55, 58.14 t (^1^*J*_РC_ 114.6 Hz), *68.41 and 69.37, 112.39 d (^3^*J*_РC_ 8.1 Hz), 115.20, 123.94 d (^1^*J*_РC_ 175.2 Hz), 129.07, 131.52 d (^3^*J*_РC_ 6.9 Hz), 131.91, 137.69 d (^3^*J*_РC_ 13.0 Hz), 156.84. ^31^P NMR (81.0 MHz, D_2_O, δ, ppm): (*9.34 + 10.20) / (12.31 + *14.96) = 2/1. Found, %: C 29.41, 29.35; H 4.64, 4.76; P 22.95, 23.15. C_10_H_18_NO_10_P_3_. Calculated, %: C 29.64, H 4.48, P 22.93.

NMR spectra of synthesized compounds are given in Supplementary materials.

### 3.2. Stability Constant Measurements and Calculations

The potentiometric titration technique using the OP-300 Radelkis potentiometer was described earlier [35]. Solutions of **8** and **9** were titrated with a standard 0.1 М NaOH solution at 298 ± 0.1 K and ionic strength of *I* = 0.1 M KCl. The initial volume of solutions was 160 mL. Titrations were performed in the range of pH 3.0–11.6 (**8**) and 3.5–11.5 (**9**). Experiments included from 33 to 52 (**8**) and from 31 to 66 (**9**) data points. The initial analytical concentrations were 0.35–0.92 mM (**8**) and 0.27–0.48 mM (**9**). The protonation constants were estimated from four titrations using the CHEMEQUI program [36] freely available on the server [37]. CHEMEQUI evaluates equilibrium constants using four different algorithms: EQ, SIMPLEX, MONTE-CARLO, and the genetic algorithm SDE [38]. Estimation of the constants was performed based on each titration. In the case of significant correlations between the protonation constants, resulting in a shift of the constants, the calculations were performed simultaneously based on several titrations. All the computational results were used to calculate the average values of the estimated full constants log*β* and their standard deviations. The average values were determined from 17 (**8**) and 12 (**9**) calculations based on several titrations and algorithms. The errors in the stepwise log*K* values are evaluated using standard deviations in estimated full equilibrium constants log*β* and an error propagation rule for several titrations and applied algorithm calculations.

Solutions of **8** or **9** with Ga(NO_3_)_3_ were titrated with NaOH under similar conditions. Titrations were performed in the range of pH 2.9–11.5 (**8**) and 3.0–12.0 (**9**). Experiments included from 31 to 60 (**8**) and from 35 to 77 (**9**) data points. The initial analytical concentrations were 0.36–0.71 mM (**8**), 0.36–0.72 mM (Ga^3+^ in experiments with **8**), 0.30–0.38 mM (**9**), and 0.31–0.45 mM (Ga^3+^ in experiments with **9**). In the calculations of stability constants of Ga^3+^ complexes with deprotonated forms of H_n_L^(5−n)–^ (for **8**, n = 0, 1,..., 5) and H_n_L^(6−n)–^ (for **9**, n = 0, 1,..., 6), the protonation constants of acids did not vary. They were taken as previously estimated in acid titration experiments. Gallium(III) forms highly stable hydroxides in water [39]. It was taken into account for obtaining unshifted estimations of the stability constants of Ga^3+^ complexes with organic ligands. The following stability constants log*β*_n_ of hydroxo-complexes in water were used in the calculations: −2.85, −7.28, −11.94, and −15.66 for the equilibria Ga^3+^ + nH_2_O = Ga^3+^(OH^−^)_n_ + nH^+^, n = 1, 2, 3, 4, respectively [40]. Stability constants of the Ga^3+^ complexes with **8** and **9** were obtained as average values from three titrations and 8 calculations for **8** and from four titrations and 8 calculations for **9**. All four CHEMEQUI algorithms were used for the calculation of stability constants.

The Hamilton’s *R*-factor (*HRF*) and the squared determination coefficient (*R*^2^_det_) were used as the agreement criteria of the proposed set of equilibrium reactions and calculated constants with the experimental data [35]. Depending on the experiment and the program algorithm, *HRF* was 0.30–0.96% (**8**), 0.46–0.92% (**9**) at the calculations of protonation constants, 0.99–1.50% (**8** + Ga^3+^), and 1.41–3.18% (**9** + Ga^3+^) at the calculations of stability constants of the Ga^3+^ complexes.

### 3.3. Radiolabelling, Stability, and Quality Control

All chemicals used for labelling were of a “reagent pure” or “extra pure” grade (Sigma-Aldrich, Panreac). The ^68^Ge/^68^Ga generators (Cyclotron Ltd., Obninsk, Russia) with activity 20 and 50 mCi were used (0–12 months after the production). The generator was eluted with 0.1 M HCl as per the manufacturer’s instruction.

Radiolabelling and evaluation of [^68^Ga]Ga-**8** and [^68^Ga]Ga-**9** stability were carried out in triplicate using procedures similar to those described in Reference [14]. In short, for labelling with ^68^Ga sodium acetate (buffering agent) solutions of various concentrations, (0.1, 0.5, or 1 M) and ^68^Ge/^68^Ga generator eluate were added to the Eppendorf tubes containing 0.1–20 μmol of **8** or **9**. pH of the mixtures was then adjusted using NaOH and HCl of pre-estimated concentrations. The reaction mixtures were stirred at 25 or 95 °C for 15–30 min. To evaluate the stability of the complexes obtained, 100 μL of a sample was added to 1000 μL of saline or fetal bovine serum. The mixtures were stirred at 37 °C for 2 h.

The content of major radiochemical impurities (colloidal and free ^68^Ga [41,42]) was determined using two strips of a radio-TLC technique. The following chromatographic systems were used: cellulose—2.4 % HCl: acetone: acetylacetone (0.8: 7: 0.5): Rf (^68^Ga_colloid_) = 0, Rf ([^68^Ga]Ga-**8**) = 0.2 ± 0.2, Rf ([^68^Ga]Ga-**9**) = 0.4 ± 0.1, Rf (^68^Ga_free_) = 0.95 ± 0.05, cellulose—HCl (1 M): methanol (2: 1): Rf (^68^Ga_colloid_) = 0, Rf ([^68^Ga]Ga-**8**) = 0.9 ± 0.1, Rf ([^68^Ga]Ga-**9**) = 0.65 ± 0.05, Rf (^68^Ga_free_) = 0.95 ± 0.05.

### 3.4. Biodistribution Studies

All experiments involving animals were performed following the ethical standards, Russian animal protection laws, and guidelines for scientific animal trials [43].

Animal studies were performed using female outbred albino rats with model pathologies. Animals with fractures (active bone callus formation) [44] had been grouped (N = 3) and [^68^Ga]Ga-**9** (100 μL per rat) was i.v. injected into the tail vein. At preselected time points (60, 120 min), animals were obtained from the experiment using partial decapitation. The organs of interest were collected, blotted dry, and weighed. Radioactivity in samples of organs/tissues was counted using a WIZARD^2^ automatic γ-counter (PerkinElmer). The results are expressed as the percentage of injected activity dose per gram (mean % ID or mean % ID/g ± SD) for each organ/tissue. For comparison, the mixture of ^68^Ge/^68^Ga generator eluate with sodium acetate solution (pH 6.5 ± 0.5, 0.18 M total acetate concentration) was also injected into animals with bone pathology.

In addition, animals with a model of aseptic inflammation were used. The site of aseptic soft tissue inflammation was modelled by intramuscular injection of 0.2 mL of turpentine into the rat pelvic limb. An acute inflammatory reaction was observed 7 days after administration. The inflammation foci were marked by swelling of the tissue, which is sharply painful on palpation. An autopsy revealed a burn of soft tissues with elements of necrosis, pronounced as a vascular pattern. [^68^Ga]Ga-**9** was studied using these animals in the same way it was done for animals with fractures. For comparison, the mixture of the ^68^Ge/^68^Ga generator eluate with sodium citrate solution (pH 5.0 ± 0.5, 0.084 M total citrate concentration) was also injected into animals.

## 4. Conclusions

The combination of aminodiphosphonic fragment with salicylic acid or its phosphonic analogue into one molecule is a promising way to develop radiopharmaceuticals. According to this technique, two new ligands with high complexation ability to gallium(III) were synthesized. Introducing phosphoryl fragment instead of carbonyl increases stability constants of the gallium(III) complexes in water. Stability constant of the Ga^3+^ complex with fully phosphorylated acid **9** (log*K*_GaL_ = 31.92 ± 0.32) significantly exceeds stability constant of Ga^3+^ complex with **8** (log*K*_GaL_ = 26.63 ± 0.24). Ligands **8** and **9** are as effective for Ga^3+^ cation binding as ethylenediamine-*N*,*N’*-diacetic-*N*,*N’*-bis(methy1enephosphonic) acid and ethylenediamine-*N*,*N*,*N’*,*N’*-tetrakis(methylenephosphonic) acid, respectively.

Both new molecules readily form ^68^Ga-complexes stable by ten-fold dilution with saline. However, in fetal bovine serum only, [^68^Ga]Ga-**9** was stable enough to be subject to biological evaluation. It was injected into rats with bone pathology and aseptic inflammation of soft tissues. In vivo studies revealed that [^68^Ga]Ga-**9** is not suitable as a bone-seeking agent, but it can be used for inflammation imaging. To an extent, as inflammation imaging, [^68^Ga]Ga-**9** is preferable over [^68^Ga]Ga-Citrate due to delayed free ^68^Ga release from the complex.

In addition, the ^68^Ga-labelling reaction with **9** was studied in detail. A correlation of acetate concentration in the reaction mixture and labelling reaction was found (up to 5 mM of **9**): the lower the acetate concentration is, the higher the labelling reaction yield is.

## Figures and Tables

**Table 1:** Stepwise protonation constants of acids **8** (H_5_L) and **9** (H_6_L) in water at 298 К and ionic strength of *I* = 0.1 M KCl ^a^.

No.	Equilibrium	log*K* ^b^		
		8	9	AEDP ^c^
1	H + L = HL	10.88 ± 0.19	11.82 ± 0.29	11.5
2	HL + H = H_2_L	9.11 ± 0.26	10.65 ± 0.43	8.58
3	H_2_L + H = H_3_L	5.65 ± 0.28	9.76 ± 0.45	5.37
4	H_3_L + H = H_4_L	3.47 ± 0.26	8.50 ± 0.45	1.50
5	H_4_L + H = H_5_L	2.99 ± 0.32	5.05 ± 0.46	
6	H_5_L + H = H_6_L		2.75 ± 0.48	

^a^ For simplicity, the charges of chemical forms in the equilibria are not indicated. ^b^ The errors in the log*K* values are evaluated using standard deviations in estimated full equilibrium constants log*β* and error propagation rule for the several titrations and applied algorithm calculations (see Experimental section). ^c^ Protonation constants of (aminoethylene)diphosphonic acid (AEDP) at 297 K and *I* = 0.2 M [24].

**Table 2:** Stepwise stability constants of the Ga^3+^ complexes with **8** (H_5_L) and **9** (H_6_L) in water at 298 К and ionic strength of *I* = 0.1 M KCl ^a^.

No.	Equilibrium	log*K*			
		8	9	EDDADPO ^b^	EDTPO ^b^
1	Ga + L = GaL	26.63 ± 0.24	31.92 ± 0.32	26.82	31.83
2	GaL + H = GaHL	8.28 ± 0.40	8.90 ± 0.61	5.10	6.65
3	GaHL + H = GaH_2_L	4.60 ± 0.43	8.28 ± 0.68	2.24	5.10
4	GaH_2_L + H = GaH_3_L	3.25 ± 0.43	8.03 ± 0.65		3.29
5	GaH_3_L + H = GaH_4_L		≈4.9		2.46

^a^ See notes for Table 2. ^b^ Stepwise stability constants of the Ga^3+^ complexes with ethylenediamine-*N*,*N’*-diacetic-*N*,*N’*-bis(methy1enephosphonic) acid (EDDADPO) and ethylenediamine-*N*,*N*,-*N’*,*N’*-tetrakis(methylenephosphonic) acid (EDTPO) at 298 K and *I* = 0.1 M KNO_3_ [25].

**Table 3:** Dynamics of distribution of [^68^Ga]Ga-**9** and [^68^Ga]Ga-acetate in rats with fractures (active bone callus formation) (mean ± SD).

Probe	[^68^Ga]Ga-Acetate		[^68^Ga]Ga-9	
Organ or Tissue	Time after Injection			
	60 min	120 min	60 min	120 min
Blood ^a^	3.0 ± 0.4	4.5 ± 0.4	2.6 ± 0.3	2.5 ± 0.0
Lung ^b^	3.3 ± 0.3	6.0 ± 0.3	3.0 ± 1.0	4.1 ± 1.4
Liver ^b^	6.8 ± 0.9	11.2 ± 0.7	4.9 ± 0.7	6.5 ± 0.5
Kidney ^b^	1.5 ± 0.1	2.6 ± 0.4	1.6 ± 0.2	1.9 ± 0.3
Intestine ^b^	4.9 ± 0.7	7.7 ± 0.5	6.4 ± 1.4	4.6 ± 1.3
Muscular tissue ^a^	0.2 ± 0.0	0.5 ± 0.0	0.3 ± 0.1	0.2 ± 0.1
Hip normal ^a^	0.8 ± 0.3	1.7 ± 0.3	0.5 ± 0.1	0.5 ± 0.1
Fraction site ^a^	1.9 ± 0.5	3.9 ± 0.9	0.6 ± 0.1	0.9 ± 0.1
Fraction site/intact bone	2.4	2.3	1.2	1.8

^a^ Specific activity accumulation was measured as a fraction (%) of the injected dose per gram of the considered organ or tissue (%ID/g). ^b^ Activity accumulation was measured as a fraction (%) of the injected dose per the considered organ (%ID/organ).

**Table 4:** Dynamics of distribution of [^68^Ga]Ga-**9** and [^68^Ga]Ga-citrate in rats with aseptic inflammation (mean ± SD).

Probe	[^68^Ga]Ga-9		[^68^Ga]Ga-Citrate	
Organ or Tissue	Time after Injection			
	60 min	120 min	60 min	120 min
Blood ^a^	5.4 ± 1.7	1.9 ± 1.0	3.1 ± 0.2	1.5 ± 0.4
Lung ^b^	2.9 ± 0.9	3.5 ± 1.2	2.6 ± 1.3	4.9 ± 2.2
Liver ^b^	7.1 ± 0.8	7.2 ± 0.9	6.9 ± 1.2	6.1 ± 0.8
Kidney ^b^	1.8 ± 0.2	1.7 ± 0.3	1.5 ± 0.3	1.5 ± 0.3
Intestines ^b^	3.8 ± 0.3	5.9 ± 0.9	5.9 ± 0.7	8.0 ± 1.5
Muscular tissue ^a^	0.3 ± 0.1	0.2 ± 0.1	0.4 ± 0.03	0.1 ± 0.1
Pathology site ^a^	1.6 ± 0.9	1.1 ± 0.5	1.1 ± 0.2	0.8 ± 0.02
Pathology site/muscular tissue	5.6	5.8	2.7	8.0

^a^ Specific activity accumulation was measured as a fraction (%) of the injected dose per gram of the considered organ or tissue (%ID/g). ^b^ Activity accumulation was measured as a fraction (%) of the injected dose per the considered organ (%ID/organ).

## Data Availability

The data presented in this study are available in Supplementary material.

## Supplementary Materials

Supplementary materials are available online. Figure S1: ^1^H and ^13^C NMR of nitrile **6a**. Figure S2: ^1^H, ^13^C and ^31^P NMR of nitrile **6b**. Figure S3: ^1^H, ^13^C and ^31^P NMR of aminodiphosphonic acid **7a**. Figure S4a: ^1^H NMR of aminodiphosphonic acid **7b**. Figure S4b: ^13^C and ^31^P NMR of aminodiphosphonic acid **7b**. Figure S5: ^1^H, ^13^C and ^31^P NMR of aminodiphosphonic acid **8**. Figure S6: ^1^H, ^13^C and ^31^P NMR of aminodiphosphonic acid **9**, Table S1: protonation constants of acids **8** and **9**. Table S2: full stability constants of the Ga^3+^ complexes with **8** and **9**.

## Sample Availability

Samples of the compounds **1**–**9** are available from the authors.

## References

[1] Roesch F., Riss P.J. (2010). The Renaissance of the 68Ge/68Ga Radionuclide Generator Initiates New Developments in 68Ga Radiopharmaceutical Chemistry. Curr. Top. Med. Chem..

[2] Velikyan I. (2014). Prospective of 68Ga-Radiopharmaceutical development. Theranostics.

[3] Baum R.P., Rosch F. (2013). Theranostics, Gallium-68, and Other radionuclides: A Pathway to Personalized Diagnosis and Treatment.

[4] Gourni E., Henriksen G., Gamez P., Caballero A.B. (2017). Metal-based PSMA radioligands. Molecules.

[5] Pillai M.R.A., Nanabala R., Joy A., Sasikumar A., Knapp F.F.R. (2016). Radiolabeled enzyme inhibitors and binding agents targeting PSMA: Effective theranostic tools for imaging and therapy of prostate cancer. Nucl. Med. Biol..

[6] Zha Z., Wu Z., Choi S.R., Ploessl K., Smith M., Alexoff D., Zhu L., Kung H.F. (2020). A New [^68^Ga]Ga-HBED-CC-Bisphosphonate as a Bone Imaging Agent. Mol. Pharm..

[7] Khawar A., Eppard E., Roesch F., Ahmadzadehfar H., Kürpig S., Meisenheimer M., Gaertner F.C., Essler M., Bundschuh R.A. (2019). Preliminary results of biodistribution and dosimetric analysis of [^68^ Ga]Ga-DOTA ZOL: A new zoledronate-based bisphosphonate for PET/CT diagnosis of bone diseases. Ann. Nucl. Med..

[8] Meckel M., Kubíček V., Hermann P., Miederer M., Rösch F. (2016). A DOTA based bisphosphonate with an albumin binding moiety for delayed body clearance for bone targeting. Nucl. Med. Biol..

[9] Meckel M., Fellner M., Thieme N., Bergmann R., Kubicek V., Rösch F. (2013). In vivo comparison of DOTA based 68Ga-labelled bisphosphonates for bone imaging in non-tumour models. Nucl. Med. Biol..

[10] Kodina G.E., Malysheva A.O., Klement’eva O.E. (2016). Osteotropic radiopharmaceuticals in Russian nuclear medicine techniques. Russ. Chem. Bull. Int. Ed..

[11] Palma E., Correia J.D.G., Campello M.P.C., Santos I. (2011). Bisphosphonates as radionuclide carriers for imaging or systemic therapy. Mol. Biosyst..

[12] Romanenko V.D., Kukhar V.P. (2012). 1-Amino-1,1-bisphosphonates. Fundamental syntheses and new developments. Arkivoc.

[13] Chmielewska E., Kafarski P. (2016). Synthetic procedures leading towards aminobisphosphonates. Molecules.

[14] Mitrofanov I.A., Maruk A.Y., Larenkov A.A., Kodina G.E., Lunev A.S., Luneva K.A., Klementyeva O.E., Tsebrikova G.S., Baulin V.E., Ragulin V.V. (2020). Evaluation of Applicability of Aminodiphosphonic Acids for the Development of Bone-Seeking 68Ga-Radiopharmaceuticals. Russ. J. Gen. Chem..

[15] Tsebrikova G., Baulin V., Ragulin V., Solov’ev V., Maruk A., Lyamtseva E., Malysheva A., Larenkov A., Zhukova M., Lunev A. (2019). New phosphonic acids as components of bone seeking radiopharmaceuticals. J. Label. Compd. Radiopharm..

[16] Rasulova N., Sagdullaev S., Arybzhanov D., Khodjibekov M., Krylov V., Lyubshin V. (2013). Optimal Timing of Bisphosphonate Administration in Combination with Samarium-153 Oxabifore in the Treatment of Painful Metastatic Bone Disease. World J. Nucl. Med..

[17] Tsebrikova G.S., Ragulin V.V., Baulin V.E., German K.E., Malysheva A.O., Klement’eva O.E., Kodina G.E., Larenkov A.A., Lyamtseva E.A., Taratonenkova N.A. (2018). 2,5-Diamino-5,5-diphosphonovaleric Acid as a Ligand for an Osteotropic 188Re Radiopharmaceutical. Russ. J. Gen. Chem..

[18] Teixeira F., Pérez A., Madden W., Hernández L., del Carpio E., Lubes V. (2016). Speciation of the ternary complexes formed between copper(II), salicylic acid and small blood serum bioligands. J. Mol. Liq..

[19] Baulin V.E., Kalashnikova I.P., Vikharev Y.B., Vikhareva E.A., Baulin D.V., Tsivadze A.Y. (2018). Phosphoryl Analogs of Salicylic Acid: Synthesis and Analgesic and Anti-Inflammatory Activity. Russ. J. Gen. Chem..

[20] Ragulin V.V. (2018). Phosphorus-Containing Aminocarboxylic Acids: XV. α,ω-Diamino-ω,ω-diphosphonoalkylcarboxylic Acids. Russ. J. Gen. Chem..

[21] Price E.W., Orvig C. (2014). Matching chelators to radiometals for radiopharmaceuticals. Chem. Soc. Rev..

[22] Delgado R., Félix V., Lima L.M.P., Price D.W. (2007). Metal complexes of cyclen and cyclam derivatives useful for medical applications: A discussion based on thermodynamic stability constants and structural data. Dalt. Trans..

[23] Popov K., Rönkkömäki H., Lajunen L.H.J. (2001). Critical evaluation of stability constants of phosphonic acids (IUPAC Technical Report). Pure Appl. Chem..

[24] Bollinger J.E., Roundhill D.M. (1993). Complexation of Indium(III), Gallium(III), Iron(III), Gadolinium(III), and Neodymium(III) Ions with Amino Diphosphonic Acids in Aqueous Solution. Inorg. Chem..

[25] Motekaitis R.J., Martell A.E. (1980). Gallium Complexes of Multidentate Ligands in Aqueous Solution. Inorg. Chem..

[26] Stein B.W., Morgenstern A., Batista E.R., Birnbaum E.R., Bone S.E., Cary S.K., Ferrier M.G., John K.D., Pacheco J.L., Kozimor S.A. (2019). Advancing Chelation Chemistry for Actinium and Other +3 f-Elements, Am, Cm, and la. J. Am. Chem. Soc..

[27] Arefyeva E., Larenkov A., Maruk A., Kodina G. (2021). Effects of Buffering Agents on Gallium-68 Speciation in Radiopharmaceutical Related Preparations. J. Label. Compd. Radiopharm..

[28] Hacht B. (2016). Gallium (III)-acetate speciation studies under physiological conditions. Open Sci. J..

[29] Clausén M., Öhman L.O., Kubicki J.D., Persson P. (2002). Characterisation of gallium(III)-acetate complexes in aqueous solution: A potentiometric, EXAFS, IR and molecular orbital modelling study. J. Chem. Soc. Dalt. Trans..

[30] Skorik N.A., Artish A.S. (1985). Stability of complexes of scandium, gallium, indium and thorium with anions of certain organic acids. Zhurnal Neorg. Khim..

[31] Petit T., Lange K.M., Conrad G., Yamamoto K., Schwanke C., Hodeck K.F., Dantz M., Brandenburg T., Suljoti E., Aziz E.F. (2014). Probing ion-specific effects on aqueous acetate solutions: Ion pairing versus water structure modifications. Struct. Dyn..

[32] Bubenshchikov V.B., Maruk A.Y., Bruskin A.B., Kodina G.E. (2016). Preparation and properties of 68Ga complexes with RGD peptide derivatives. Radiochemistry.

[33] Lunyov A.S., Clement’eva О.Е., Lunyova К.А., Zhukova M.V., Malysheva А.О. (2017). Qualitative and quantitative comparisons of bone PET-imaging using 68Ga-oxabiphor and Na18F. Saratov J. Med. Sci. Res..

[34] Lunev A.S., Larenkov A.A., Petrosova K.A., Klementyeva O.E., Kodina G.E. (2016). Fast PET imaging of inflammation using ^68^Ga-citrate with Fe-containing salts of hydroxy acids. EJNMMI Radiopharm. Chem..

[35] Tsebrikova G.S., Barsamian R.T., Solov V.P., Kudryashova Z.A., Baulin V.E., Wang Y.J., Tsivadze A.Y. (2018). Complexation of gallium(III) nitrate with 1,4,7,10-tetraazacyclododecane-1,4,7,10-tetrakis(methylenephosphonic acid). Russ. Chem. Bull..

[36] Solov’ev V.P., Tsivadze A.Y. (2015). Supramolecular complexes: Determination of stability constants on the basis of various experimental methods. Prot. Met. Phys. Chem. Surfaces.

[37] Solov’ev V.P. The CHEMEQUI Program for Computations of Equilibrium Constants and Related Quantities from Experimental Results of UV–Vis, IR and NMR Spectroscopy, Calorimetry, Potentiometry and Conductometry. http://vpsolovev.ru/programs/chemequi/.

[38] Ali M., Pant M., Abraham A. (2012). A simplex differential evolution algorithm: Development and applications. Trans. Inst. Meas. Control.

[39] Wood S.A., Samson I.M. (2006). The aqueous geochemistry of gallium, germanium, indium and scandium. Ore Geol. Rev..

[40] Benézéth P., Diakonov I.I., Pokrovski G.S., Dandurand J.L., Schott J., Khodakovsky I.L. (1997). Gallium speciation in aqueous solution. Experimental study and modelling: Part 2. Solubility of α-GaOOH in acidic solutions from 150 to 250°C and hydrolysis constants of gallium (III) to 300 °C. Geochim. Cosmochim. Acta.

[41] Larenkov A.A., Maruk A.Y., Kodina G.E. (2018). Intricacies of the Determination of the Radiochemical Purity of ^68^Ga Preparations: Possibility of Sorption of Ionic ^68^Ga Species on Reversed-Phase Columns. Radiochemistry.

[42] Maruk A.Y., Larenkov A.A. (2020). Determination of ionic ^68^Ga impurity in radiopharmaceuticals: Major revision of radio-HPLC methods. J. Radioanal. Nucl. Chem..

[43] GOST 33216-2014 (2014). Guidelines for Accommodation and Care of Animals. Species-Specific Provisions for Laboratory Rodents and Rabbits.

[44] Moiseenko V.M., Blinov N.N. (1996). Sovremennaya Taktika Lecheniya Bol’nykh so Zlokachestvennymi Novoobrazovaniyami s Metastazami v Kosti: Posobie Dlya Vrachei (Modern Tactics of Treating Patients with Malignant Neoplasms with Bone Metastases: A Manual for Doctors, in Russian).

