# Supplementary material for: Synthesis, Complexation Properties, and Evaluation of New Aminodiphosphonic Acids as Vector Molecules for 68Ga Radiopharmaceuticals
Source: Molecules. 2021 Apr 18;26(8):2357. doi: 10.3390/molecules26082357 (PMC8073962; doi:10.3390/molecules26082357)
Supplement: Supplementary file 1 [file molecules-26-02357-s001.zip › molecules-1146724-supplementary.pdf]

Supplementary materials

Synthesis, Complexation Properties, and Evaluation of New Aminodiphosphonic Acids as Vector Molecules for ^{68}Ga Radiopharmaceuticals

Alesya Ya. Maruk[1], Valery V. Ragulin[2], Iurii A. Mitrofanov[1], Galina S. Tsebrikova[3,*], Vitaly P. Solov'ev[3], Alexandr S. Lunev[1], Kristina A. Lunyova[1], Olga E. Klementyeva[1], Vladimir E. Baulin[2], Galina E. Kodina[1], and Aslan Yu. Tsivadse[3]

[1]*Department of Radiation Medical Technologies, State Research Center – Burnasyan Federal Medical Biophysical Center of Federal Medical Biological Agency, Moscow, Russian Federation*

[2]*Laboratory of Organophosphorus Compounds, Institute of Physiologically Active Compounds, Russian Academy of Sciences, Chernogolovka, Russian Federation*

[3]*Laborotary of Novel Physicochemical Problems, Frumkin Institute of Physical Chemistry and Electrochemistry, Russian Academy of Sciences, Moscow, Russian Federation*

Contents **Page**

Figure S1	^{1}H and ^{13}C NMR of nitrile **6a**	2
Figure S2	^{1}H, ^{13}C and ^{31}P NMR of nitrile **6b**	3
Figure S3	^{1}H, ^{13}C and ^{31}P NMR of aminodiphosphonic acid **7a**	4
Figure S4a	^{1}H NMR of aminodiphosphonic acid **7b**	5
Figure S4b	^{13}C and ^{31}P NMR of aminodiphosphonic acid **7b**	6
Figure S5	^{1}H, ^{13}C and ^{31}P NMR of aminodiphosphonic acid **8**	7
Figure S6	^{1}H, ^{13}C and ^{31}P NMR of aminodiphosphonic acid **9**	8
Table S1	Protonation constants of acids **8** and **9**	9
Table S2	Full stability constants of the Ga^{3+} complexes with **8** and **9**	9

Figure S1. ^{1}H and ^{13}C NMR of nitrile **6a**.

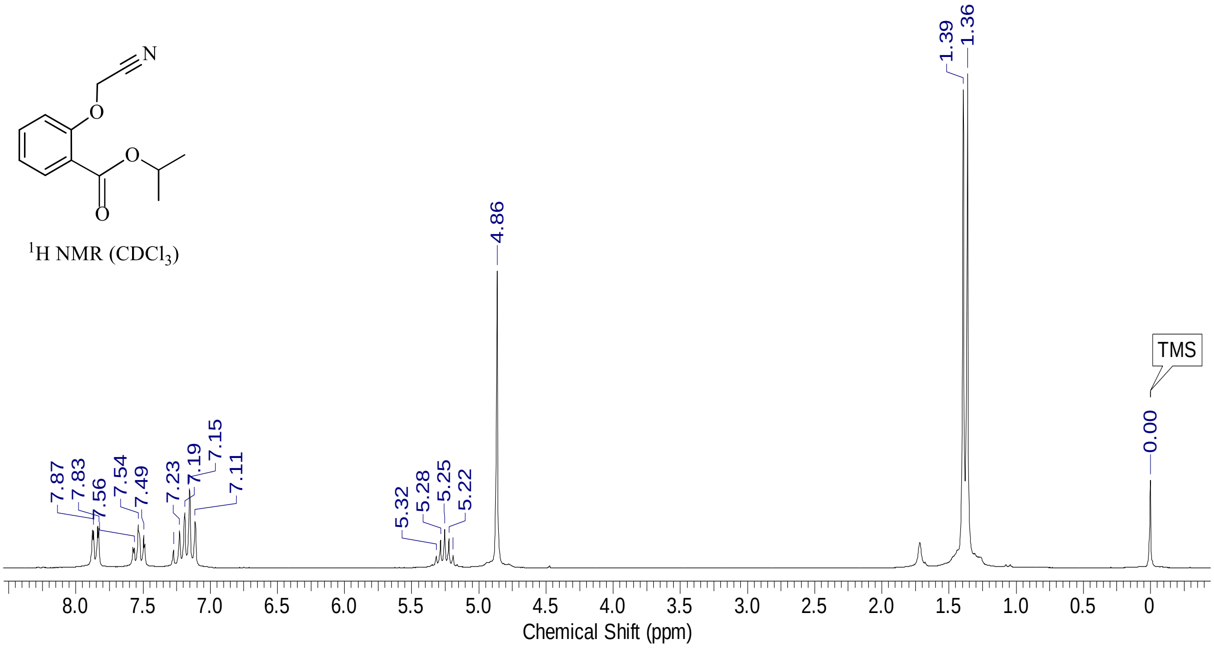

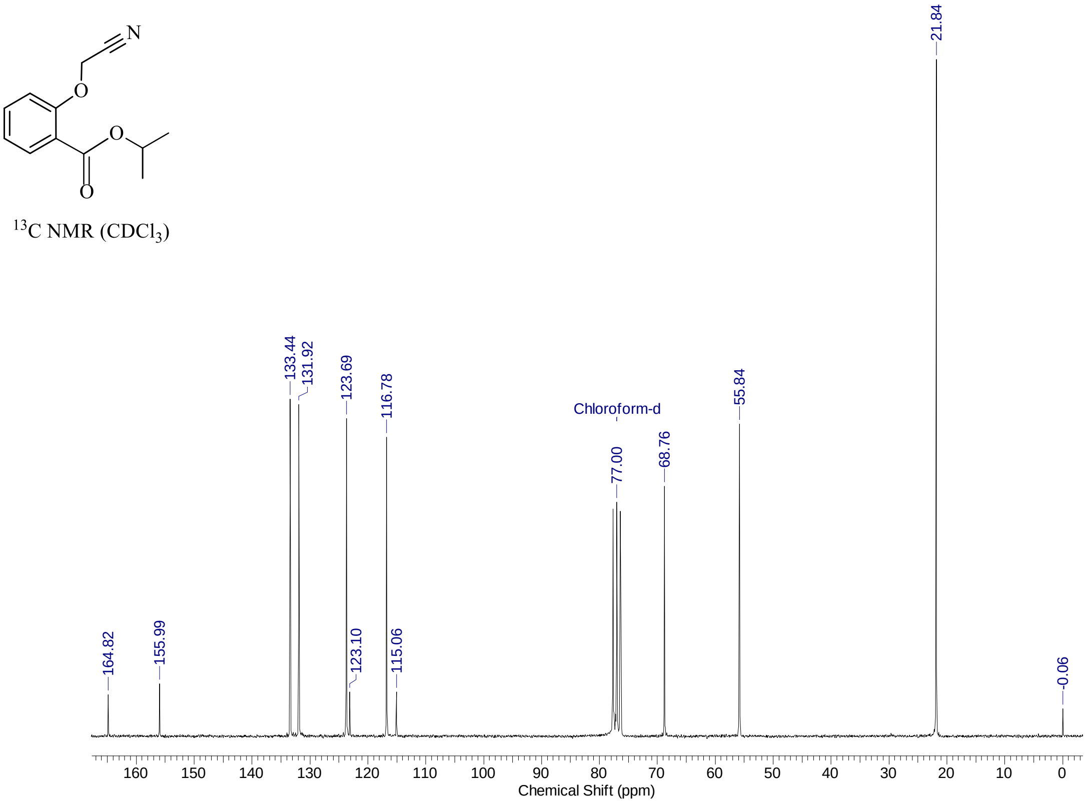

Figure S2. ^{1}H, ^{13}C and ^{31}P NMR of nitrile **6b**.

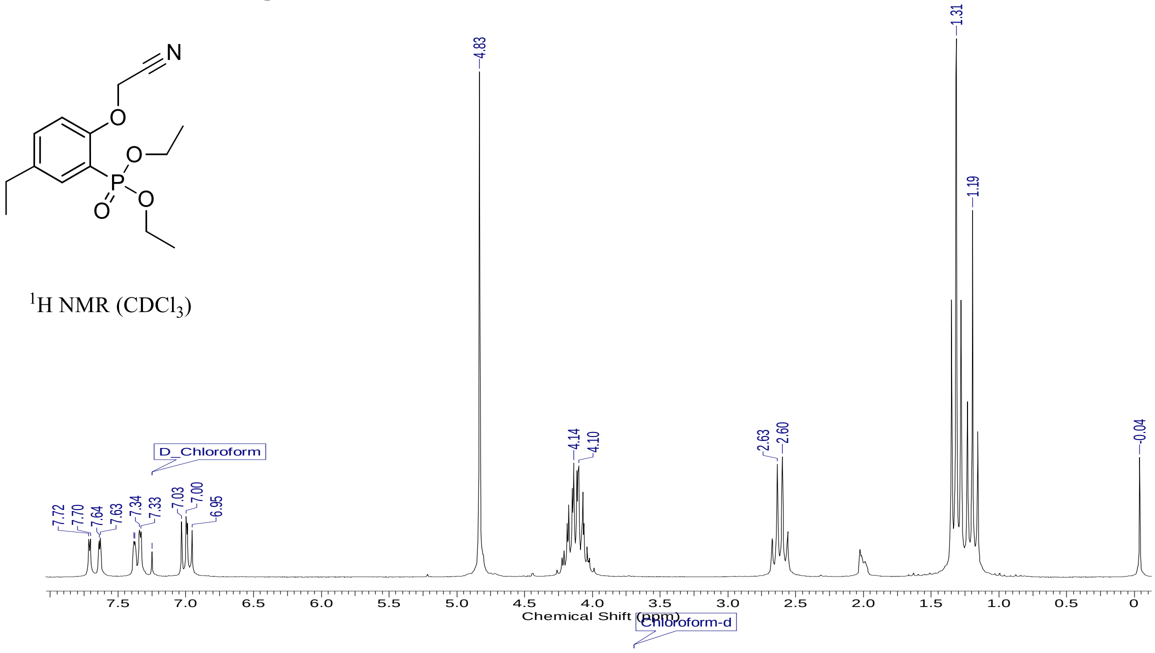

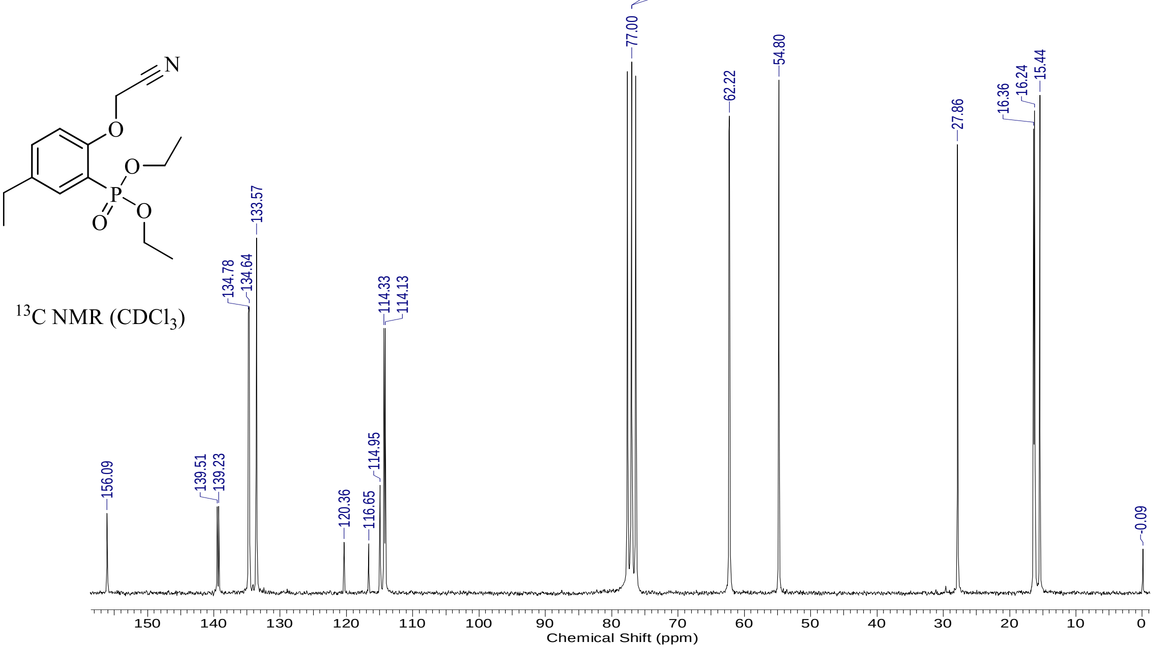

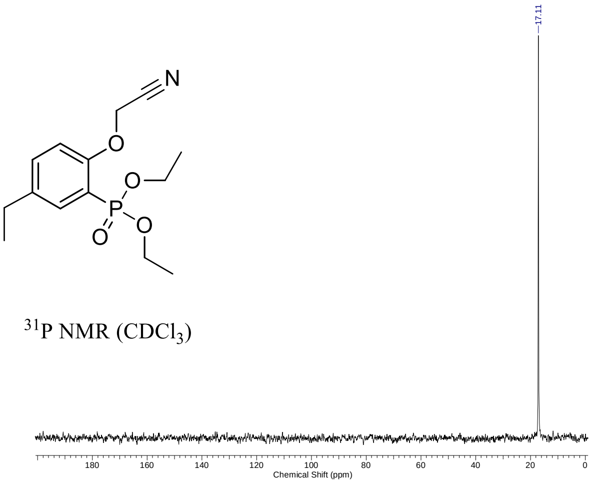

Figure S3. ^{1}H, ^{13}C and ^{31}P NMR of aminodiphosphonic acid **7a**.

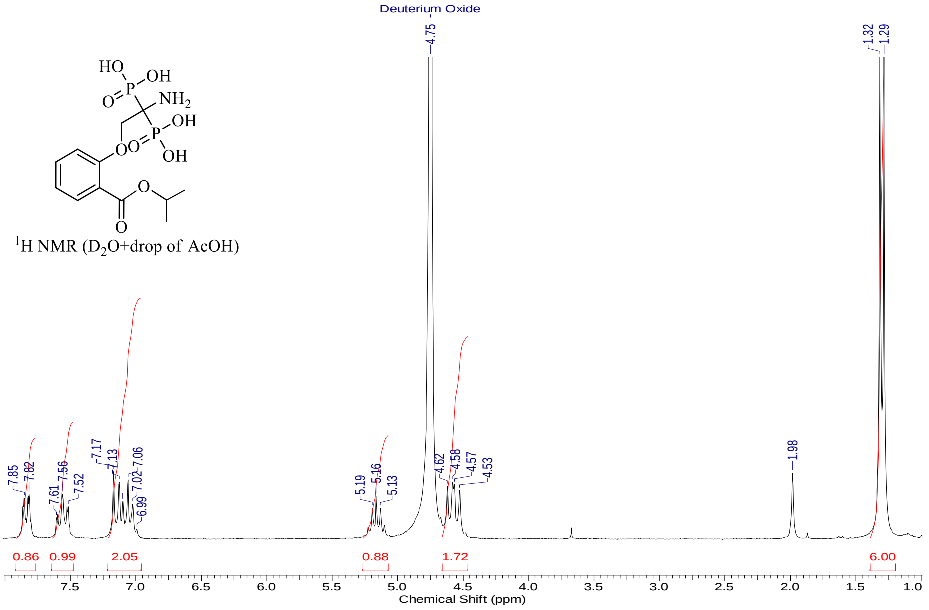

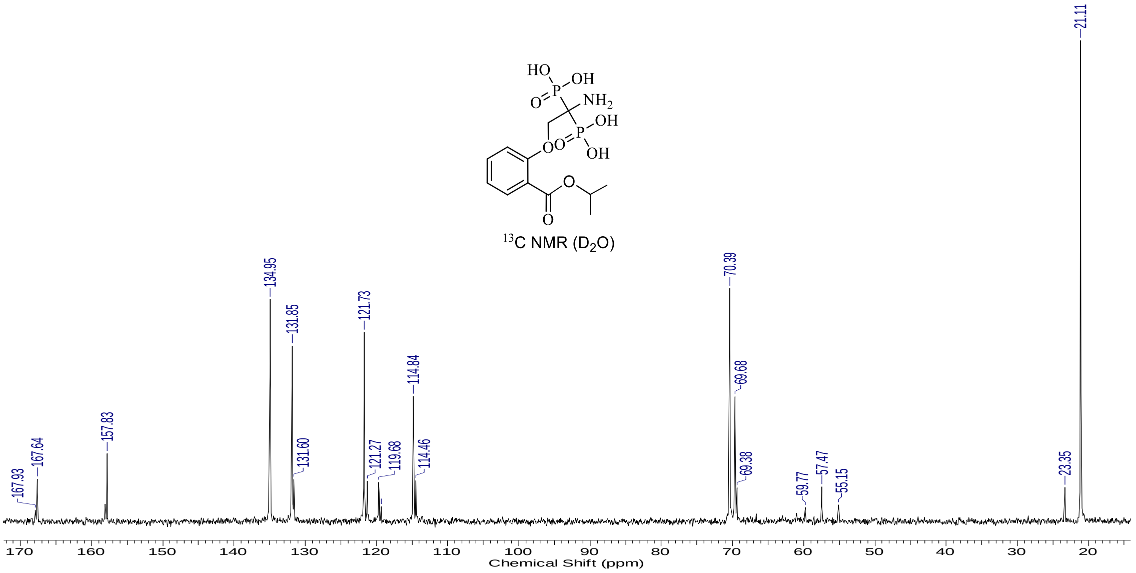

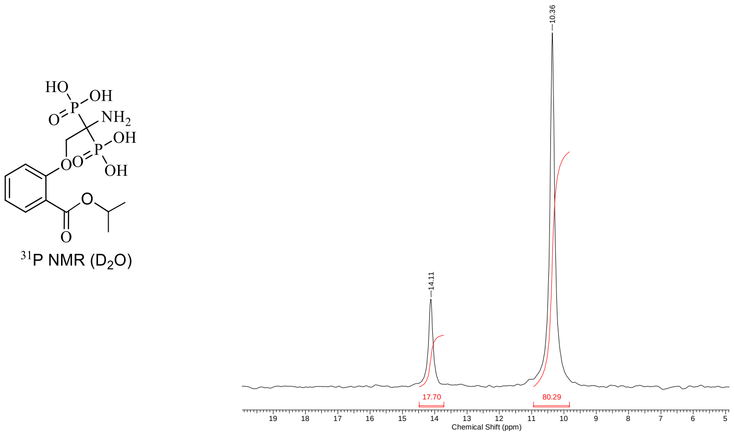

Figure S4a. ^{1}H NMR of aminodiphosphonic acid **7b**.

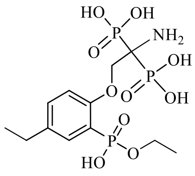

^{1}H NMR (D_2O)

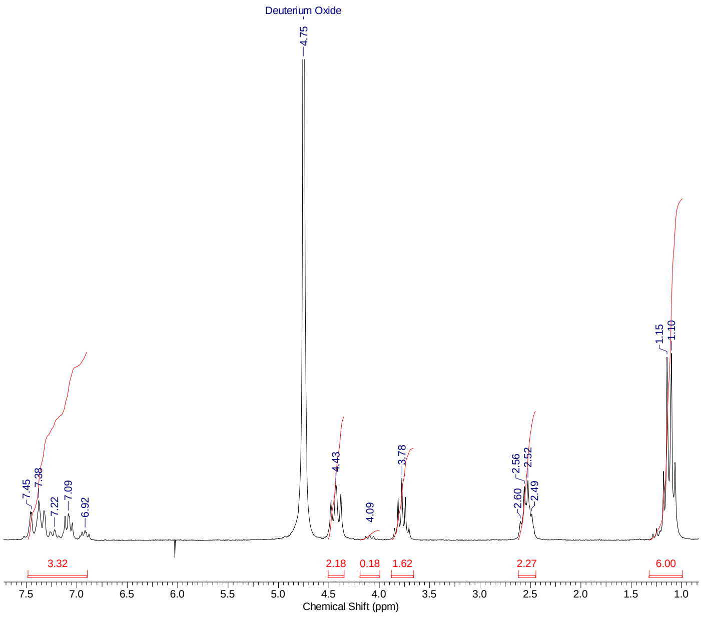

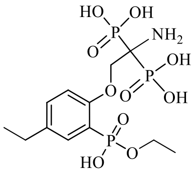

^{1}H NMR (DMSO-d_6)

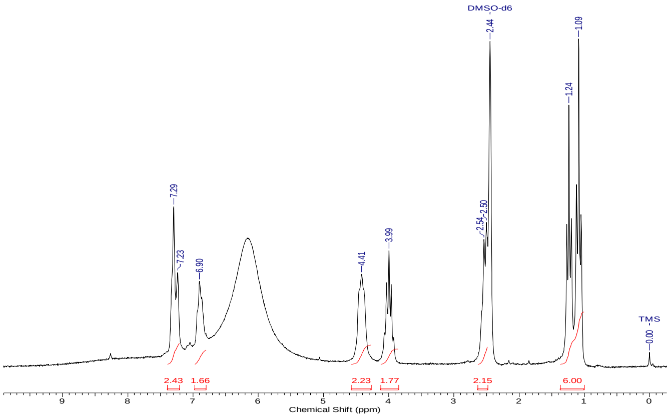

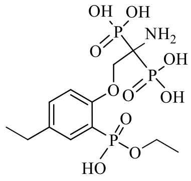

^{1}H NMR (DMSO-d_6 + drop of TFA)

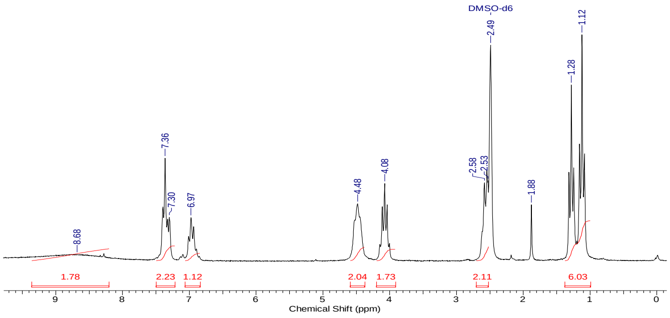

Figure S4b. ^{13}C and ^{31}P NMR of aminodiphosphonic acid **7b**.

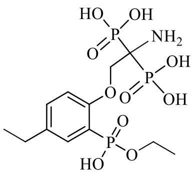

^{13}C NMR (D_2O)

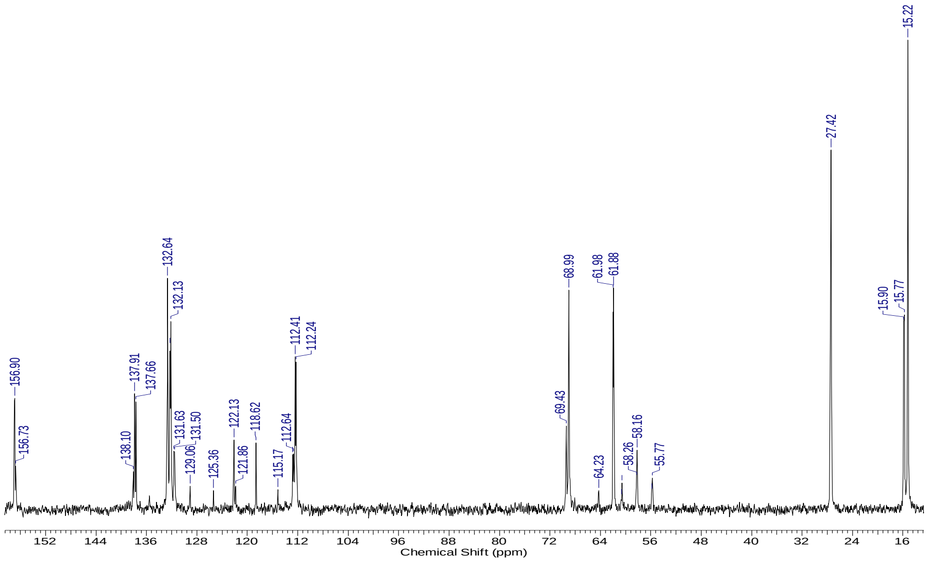

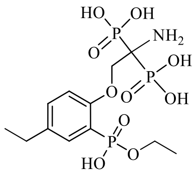

^{31}P NMR (DMSO d_6)

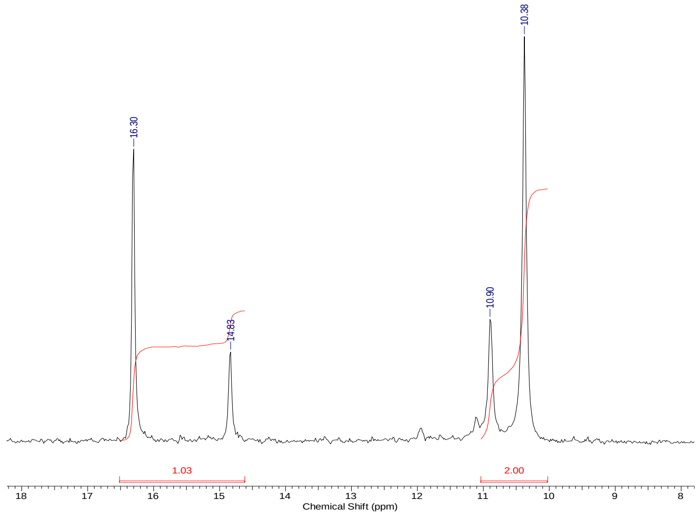

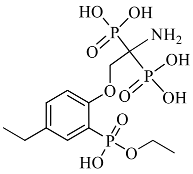

^{31}P NMR (D_2O)

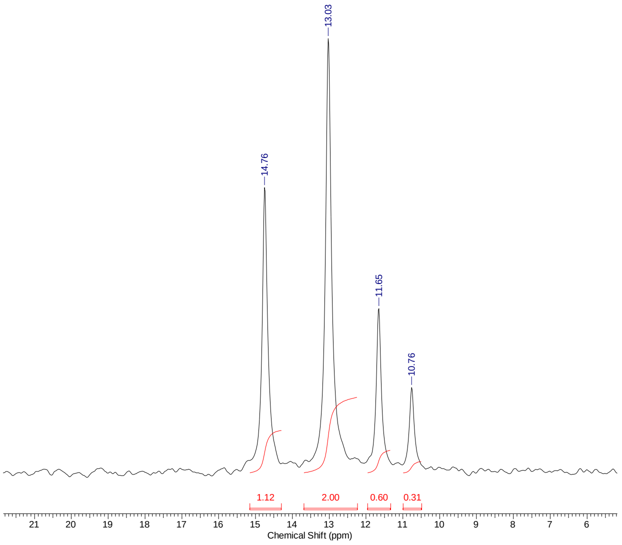

Figure S5. ^{1}H, ^{13}C and ^{31}P NMR of aminodiphosphonic acid **8**.

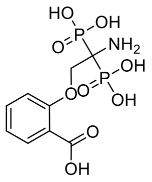

1

NMR ^{1}H (D_2O, pH~10)

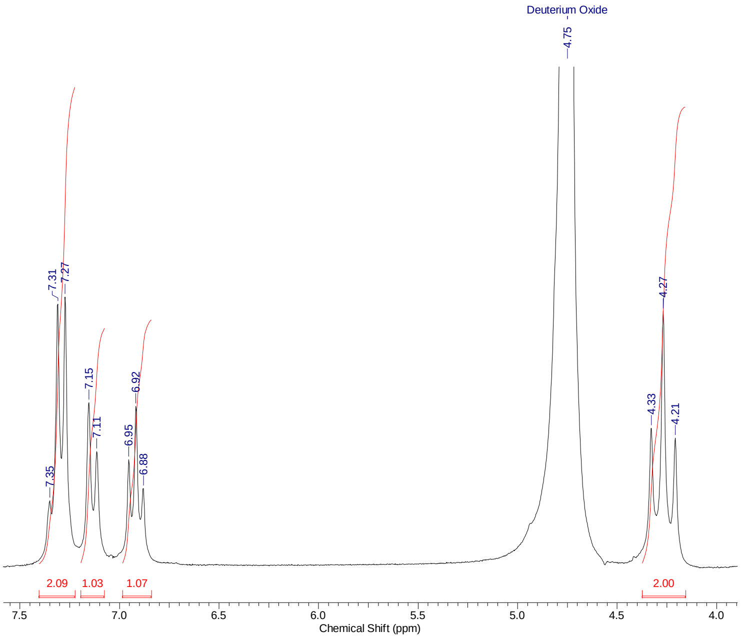

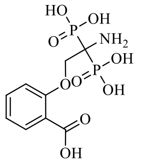

1

NMR ^{13}C (D_2O, pH~10)

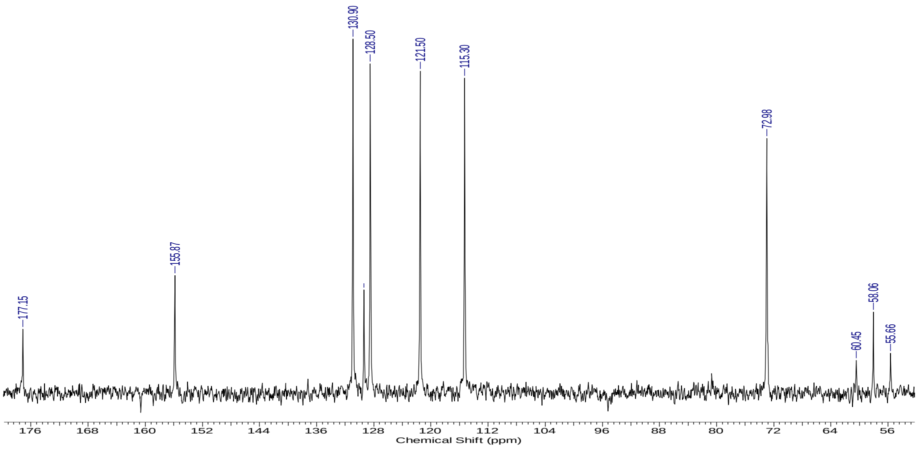

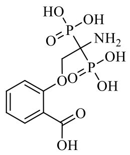

1

NMR ^{31}P (D_2O, pH~5)

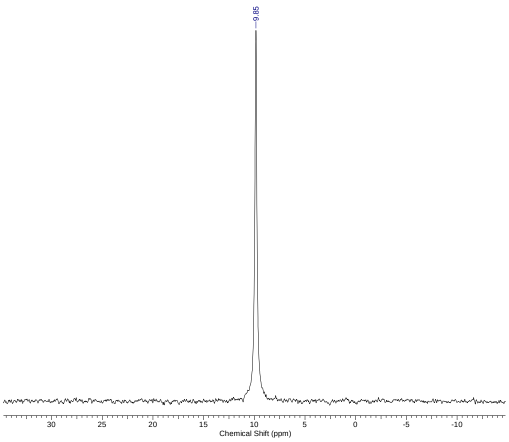

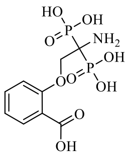

1

NMR ^{31}P (D_2O, pH~10)

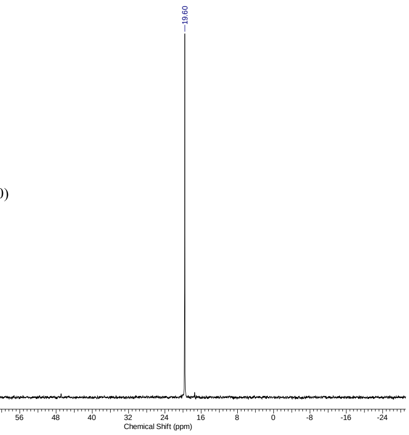

Figure S6. ^{1}H, ^{13}C and ^{31}P NMR of aminodiphosphonic acid **9**.

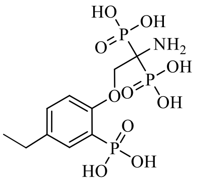

2

^{1}H NMR (D_2O, pH~5)

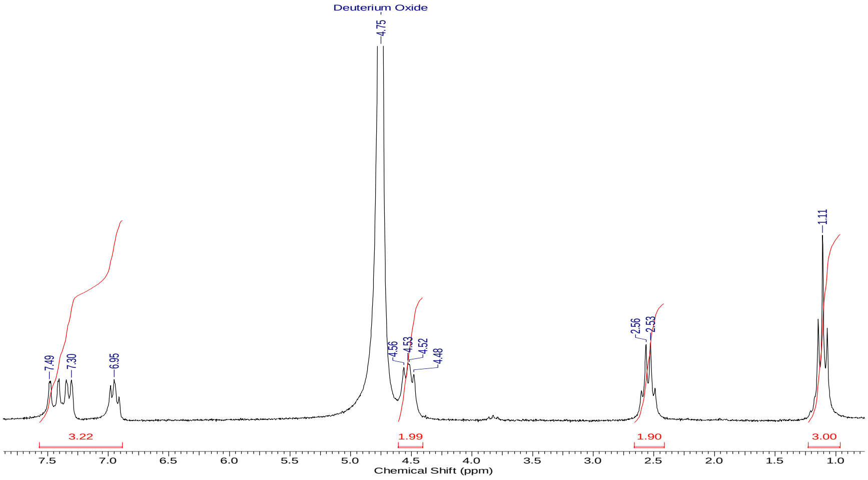

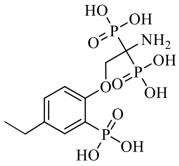

2

^{13}C NMR (D_2O, pH~5)

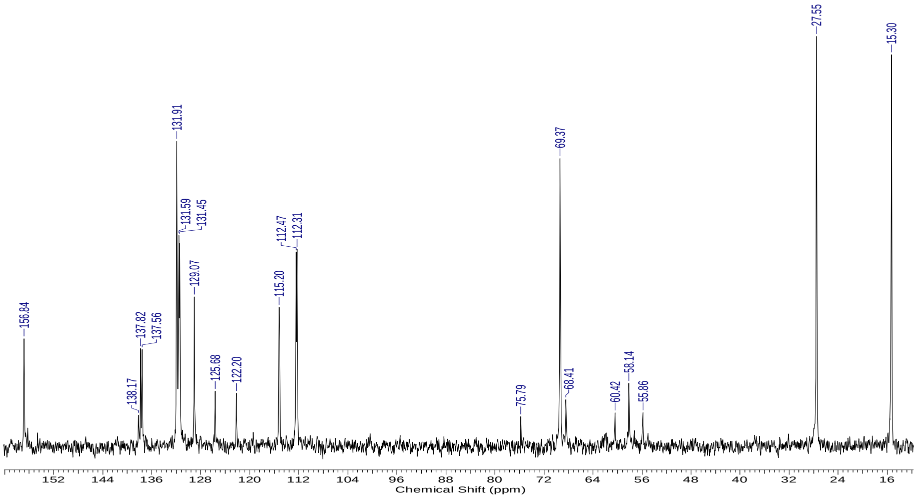

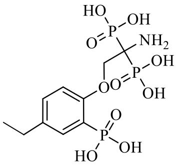

2

^{31}P NMR (D_2O, pH~5)

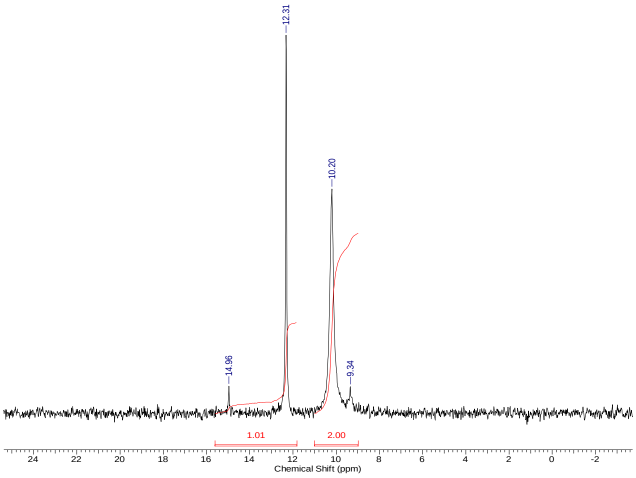

The presence of the second form of acid **9** is likely determined by the syn- or anti-orientation of the methyl group of the ethyl radical and the aminodiphosphonic function in molecule **9**, which is likely why the ratio of conformers, in this case, is ~ 1:10.

Table S1. Protonation constants of acids **8** (H_5L) and **9** (H_6L) in water at 298 K and ionic strength of I = 0.1 M KCl [a].

No.	Equilibrium	logβ [b]			
		**8**	**9**	MAMDP [c]	AEDP [c]
1	L + H = HL	10.88 ± 0.19	11.82 ± 0.29	13.4	11.5
2	L + 2H = H_2L	19.99 ± 0.18	22.47 ± 0.32	21.95	20.08
3	L + 3H = H_3L	25.64 ± 0.21	32.23 ± 0.32	26.81	25.45
4	L + 4H = H_4L	29.10 ± 0.15	40.73 ± 0.32	27.87	26.95
5	L + 5H = H_5L	32.09 ± 0.28	45.78 ± 0.33		
6	L + 6H = H_6L	-	48.52 ± 0.34		

[a] For simplicity, the charges of chemical forms in the equilibria are not indicated.
[b] logβ are full equilibrium constants, the errors in the lgβ values are evaluated by error propagation rule for the several titrations and applied algorithm calculations (see Experimental section)
[c] Protonation constants of similar known ligands [22]: (dimethylamino)methylenediphosphonic acid (MAMDP) and (aminoethylene)diphosphonic acid (AEDP), at 297 K and I = 0.2 M.

Table S2. Full stability constants of the Ga^{3+} complexes with **8** (H_5L) and **9** (H_6L) in water at 298 K and ionic strength of I = 0.1 M KCl [a].

No.	Equilibrium	logβ				
		**8**	**9**	GDPO [b]	EDDADPO [b]	EDTPO [b]
1	Ga + L = GaL	26.63 ± 0.24	31.92 ± 0.32	21.93	26.82	31.83
2	Ga + H +L = GaHL	34.91 ± 0.32	40.82 ± 0.51	26.02	31.92	38.48
3	Ga + 2H +L = GaH_2L	39.51 ± 0.29	49.10 ± 0.45	28.53	34.16	43.58
4	Ga + 3H +L = GaH_3L	42.76 ± 0.32	57.13 ± 0.46			46.87
5	Ga + 4H + L = GaH_4L	-	≈ 62			49.33

[a] See notes for Table S1.
[b] Full stability constants of the Ga^{3+} complexes with known multidentate ligands containing two or four phosphonic groups [23]: glycine-*N*,*N*-bis(methylenephosphonic) acid (GDPO), ethylenediamine-*N*,*N'*-diacetic-N,N'-bis(methylenephosphonic) acid (EDDADPO), and ethylenediamine-*N*,*N*,*N'*,*N'*-tetrakis(methylenephosphonic) acid (EDTPO), at 298 K and I = 0.1 M KNO_3.
